# Permutation Entropy: Enhancing Discriminating Power by Using Relative Frequencies Vector of Ordinal Patterns Instead of Their Shannon Entropy

**DOI:** 10.3390/e21101013

**Published:** 2019-10-18

**Authors:** David Cuesta-Frau, Antonio Molina-Picó, Borja Vargas, Paula González

**Affiliations:** 1 Technological Institute of Informatics, Universitat Politècnica de València, 03801 Alcoi Campus, Spain; antoniomolina@disca.upv.es; 2 Innovatec Sensorización y Comunicación S.L., Avda. Elx, 3, 03801 Alcoi, Spain; 3 Department of Internal Medicine, Móstoles Teaching Hospital, Móstoles, 28935 Madrid, Spain; borjavargas1@gmail.com (B.V.); paulagain84@gmail.com (P.G.)

**Keywords:** permutation entropy, hidden Markov models, *k*-means clustering, signal classification, relative frequency estimation, feature selection, body temperature

## Abstract

Many measures to quantify the nonlinear dynamics of a time series are based on estimating the probability of certain features from their relative frequencies. Once a normalised histogram of events is computed, a single result is usually derived. This process can be broadly viewed as a nonlinear ${I\;R}^{n}$ mapping into IR, where *n* is the number of bins in the histogram. However, this mapping might entail a loss of information that could be critical for time series classification purposes. In this respect, the present study assessed such impact using permutation entropy (PE) and a diverse set of time series. We first devised a method of generating synthetic sequences of ordinal patterns using hidden Markov models. This way, it was possible to control the histogram distribution and quantify its influence on classification results. Next, real body temperature records are also used to illustrate the same phenomenon. The experiments results confirmed the improved classification accuracy achieved using raw histogram data instead of the PE final values. Thus, this study can provide a very valuable guidance for the improvement of the discriminating capability not only of PE, but of many similar histogram-based measures.

## 1. Introduction

Classification of time series is one of the main applications of pattern recognition and machine learning [1]. From a collection of features extracted from each time series, a varied and diverse set of methods have been proposed to assign a class label to a group considered homogeneous using a certain dissimilarity criterion [2]. These methods have been applied to any scientific or technological field where a temporal sequence of data is generated [3,4,5,6,7,8].

The present paper is focused specifically on the classification of time series using signal complexity features. Such features exhibit a very high discriminating power, and that is why they have been successfully employed in many applications [9,10,11]. However, there are ongoing efforts to further improve this discriminating power with new complexity estimation algorithms or by tweaking current ones [12,13,14,15,16,17], as is the case in this paper.

Many measures or methods have been described in the scientific literature so far to quantify time series complexity, irregularity, uncertainty, predictability or disorder, among other similar terms devised to characterise their dynamical behaviour [18]. The present study deals specifically with the concept of entropy, one of the previous terms related to expected information, and often employed as a single distinguishing feature for time series classification. Entropy, as an information gauge, has also been defined in many forms, with higher entropy values accounting for higher uncertainty.

The concept of entropy was first applied in the context of thermodynamics [19]. A few decades later, other scientific and technological areas adapted and customised this concept to information theory [20]. The Shannon entropy introduced in [21] soon became one of the most used and successful of these measures. In its discrete and generic form, it is given by

(1) $$h_{S}=-\sum_{\omega \in \Omega }p\left(\omega \right)\log p\left(\omega \right)$$

where ω is a random variable with probability distribution $p\left(\omega \right)$ over the probability space Ω, and log is usually a base 2 logarithm, although natural logarithms are fairly common too.

For the focus of interest of the present paper, classification of time series, Equation (1) must be customised for sequences of numerical values. Assuming a stationary ergodic time series, x, containing amplitude values of a continuous process with a sampling period of *T*, this time series can be written as the discrete time vector $x=\left\{x[0T],x[1T],x[2T],\ldots ,x[(N-1)T]\right\}$, or, in a more compact and simple form, as $x=\left\{x_{0},x_{1},x_{2},\cdots ,x_{N-1}\right\}$, with length *N*. At this point, Equation 1 could be applied to the samples in x. However, as in most real applications *p* is unknown and *N* is finite, an estimation, $\hat{p}$, based on event counting or relative frequency has to be used instead.

There are many possible available choices as for the event type related to x from which to obtain $\hat{p}$. We adopted a block approach, but instead of directly using samples or subsequences from the time series, we used associated ordinal patterns (more details in Section 2.1) from consecutive *m* overlapping windows, $x_{i}^{m}=\left\{x_{i},x_{i+1},x_{i+2},\cdots ,x_{i+m-1}\right\}$. This way, the number of different blocks is finite and known in advance, and the relative frequencies can be estimated robustly provided the length of the blocks *m* is significantly shorter than the length of the time series, m<<N [18]. If each possible ordinal pattern is referred to as $\Pi_{j}^{m}$, with 0≤j<m!, Equation (1) takes the form

(2) $${\hat{h}}_{S}^{m}=-\sum_{j=0}^{m!-1}\hat{p}\left(\Pi_{j}^{m}\right)\log\hat{p}\left(\Pi_{j}^{m}\right)$$

where $\hat{p}\left(\Pi_{i}^{m}\right)$ is the relative frequency of the ordinal pattern $\Pi_{i}^{m}$. For example, for subsequences of length 3, with sample position indices ranging from 0 to 2, the ordinal patterns $\Pi_{j}^{3}$ that can be found are (0,1,2), (0,2,1), (1,0,2), (1,2,0), (2,1,0) and (2,0,1).

**Figure 1:**
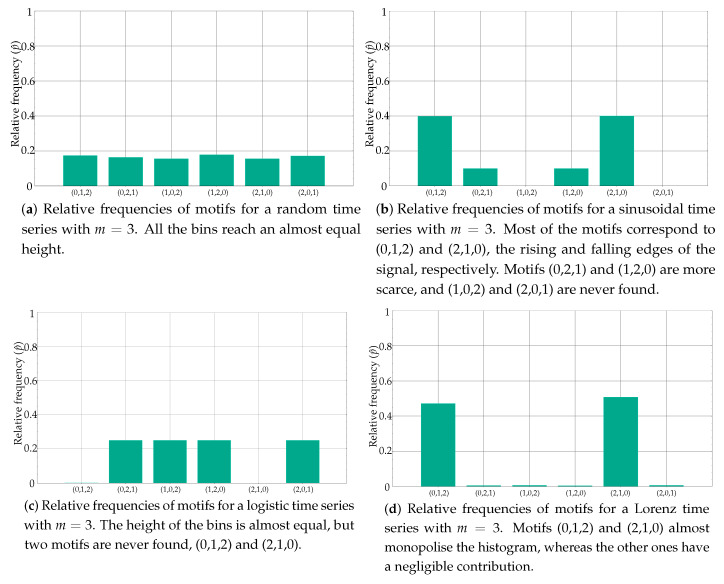
Examples of histograms from well-known synthetic time series. Some features of these series are clearly reflected in the histograms, with a clear correlation between the distribution of the motifs and the determinism degree of the records.

Figure 1 depicts the histograms for a few popular synthetic time series, as an example of the distribution of these relative frequencies. Specifically, the histogram in Figure 1a belongs to a random time series with a uniform distribution, as can be inferred from the equality of the bins. At the opposite end of the spectrum of randomness, Figure 1b shows the histogram of a sinusoidal time series, with a more polarised distribution of motifs, some of them forbidden (zero probability) [22]. Figure 1c,d shows the histograms of other time series with a different degree of determinism, a Logistic (coefficient 3.5) and a Lorenz (parameters 10, 8/3 and 28) time series, respectively [23,24]. When computing the associated entropy to the relative frequencies shown in Figure 1, the single value obtained will be arguably very different in each case.

However, the mapping of relative frequencies into a single scalar (in the sense of a size 1 vector) may entail a significant information loss, as there are infinite vectors of estimated probabilities $\hat{p}=\left\{\hat{p}\left(\Pi_{0}^{m}\right),\hat{p}\left(\Pi_{1}^{m}\right),\ldots ,\hat{p}\left(\Pi_{m!-1}^{m}\right)\right\}$ that can yield the same ${\hat{h}}_{S}^{m}$. One of the most successful applications of entropy statistics is time series classification, and this information loss could have a detrimental impact on the accuracy achieved in these applications.

The present paper is aimed at characterising those situations where a single entropy measure may fail in providing discriminating information due to histogram compensation. This study will be based on a specific Shannon entropy embodiment: permutation entropy (PE) [25]. Despite the good results achieved using this measure, there are still cases where the histogram differences are lost when computing the final PE value. We illustrate this situation with synthetic records generated using hidden Markov models (HMM), and with real body temperature records that exhibit this same behaviour. We then propose a different approach based on a clustering scheme that uses all the relative frequencies as time series features to overcome this problem. The results for both types of experimental time series demonstrate how critical this information loss may become, and how the proposed clustering method can solve it.

There is no similar study in the scientific literature, as far as we know. However, there are a few works that could use a multidimensional classification approach as ours. For example, it is worth describing the approach taken by [26]. This paper presents a method to classify beat-to-beat intervals data series from healthy and congestive heart failure patients using classical RR features, a symbolic representation and ordinal pattern statistics. It first considers each feature individually, and then a combination of two using a linear support vector machine. The main difference with the approach described in the present paper is that the method is fully supervised, and customised for a specific biomedical record, without taking into account the full picture of the PE context.

Another key concept that will play an important role in this work is the concept of forbidden ordinal pattern [18]. In a general sense, we consider a forbidden pattern with an ordinal pattern with a relative frequency of 0 and a forbidden transition i→j, the impossibility to generate an ordinal pattern *j* if the previous one was an ordinal pattern *i*. Forbidden patterns have already demonstrated their usefulness to detect determinism in time series [27,28], and have even been used for classification purposes already [22,23], as additional distinguishing features. Specifically, the present study will use forbidden transitions as a tool to generate synthetic time series including forbidden patterns or certain histogram distributions.

## 2. Materials and Methods

### 2.1. Permutation Entropy

PE is the Shannon entropy of the estimated probabilities of ordinal patterns found in input time series x [25]. To obtain these ordinal patterns, consecutive subsequences of length *m* and commencing at sample *i* have to be drawn from x. Formally, these subsequences can be defined as $x_{i}^{m}=\left\{x_{i},x_{i+1},\ldots ,x_{i+m-1}\right\}$, where the index *i* ranges from 0 to N-m, with *N* being the total length of x. Initially, when the subsequences are first extracted from the time series, the order of $x_{i},x_{i+1},\ldots ,x_{i+m-1}$ can be any, but the indices of the samples are always taken as 0,1,…,m-1 by default. When $x_{i},x_{i+1},\ldots ,x_{i+m-1}$ are sorted in ascending order, the resulting new order is translated to the vector of indices. This new order of indices is considered the ordinal pattern or motif that represents $x_{i}^{m}$. We will refer to this vector as $\pi_{i}^{m}$. For example, if $x_{i}^{3}=\left\{1.7,-0.3,2.5\right\}$, the associated motif is (1,0,2), as the minimum, −0.3, was located at index 1, the next value in ascending order, 1.7, at index 0, and the maximum value, 2.5, at index 2. Therefore, $\pi_{i}^{3}=\left(1,0,2\right)$. The corresponding counter (histogram bin) of motif $\left(1,0,2\right)$, $\Pi_{j}^{3}$, will therefore be increased, $c_{\Pi_{j}^{3}}\leftarrow c_{\Pi_{j}^{3}}+1$ (supposing $\Pi_{j}^{3}=\left(1,0,2\right)$). For simplification purposes, *m* will be 3 in all the tests conducted.

This process is repeated for all the possible subsequences in x, and the final relative frequency of each *j*-th motif is computed, obtaining $\hat{p}$ using the normalised histogram of such motifs. Thus, $\hat{p}$ specifically becomes in PE $\hat{p}=\left\{\hat{p}\left(\Pi_{0}^{m}\right),\hat{p}\left(\Pi_{1}^{m}\right),\hat{p}\left(\Pi_{2}^{m}\right),\cdots ,\hat{p}\left(\Pi_{m!-1}^{m}\right)\right\}$, as described in the previous section. Finally, $\hat{p}$ is used to calculate PE as

(3) $$\mathrm{PE}\left(x,m,N\right)=-\sum_{k=0}^{m!-1}\hat{p}\left(\Pi_{k}^{m}\right)\log\hat{p}\left(\Pi_{k}^{m}\right),\forall \hat{p}\left(\Pi_{k}^{m}\right)>0$$

which essentially coincides with Equation (2). This entropy can be further normalised by the embedded dimension *m*, enabling the PE comparison for different *m* and by $\log\left(m!\right)$. There are versions based on Reny or Tsallis entropies instead [14].

### 2.2. Clustering Algorithm

The objective of a clustering algorithm is to find in an unsupervised way natural groups in any dataset according to certain features and a dissimilarity measure or metric [29]. Specifically, the purpose of using a clustering algorithm in this study is to separate automatically the classes in the experimental datasets based on the relative frequencies of motifs.

The selection of a specific method of clustering among the thousands available depends on the features of the input data, the achievable accuracy, implementation issues, and computational cost, among other possible requirements [30]. In any case, the goal is to minimise the intracluster distance or dissimilarity between objects (time series) and maximise the intercluster one [31].

We specifically chose the *k*-means clustering algorithm [32]. This is probably the most widely used and known clustering algorithm, and, after more than 50 years since its introduction [33], it remains fully in use, in its original form or in its many spawned evolutions [34]. It is also simple to implement and available in many software tools and libraries. We have also previously used this method successfully [4,35,36,37].

The input to the clustering algorithm in this case is a set of time series defined by their features, their estimated probabilities $\left\{\hat{p}\left(\Pi_{0}^{m}\right),\hat{p}\left(\Pi_{1}^{m}\right),\hat{p}\left(\Pi_{2}^{m}\right),\cdots ,\hat{p}\left(\Pi_{m!-1}^{m}\right)\right\}$. The dissimilarity *d* between two time series, *i* and *j*, will correspond to the distance between their two associated estimated probabilities vectors $d\left({\hat{p}}_{i},{\hat{p}}_{j}\right)$. This distance will be based on the Euclidean metric [38]. The number of clusters is set in advance as 2 (to account for the two classes included in all the experimental datasets), avoiding one of the main problems in many clustering algorithms [39].

Before the *k*-means iteration takes place, it is necessary to obtain an initial set of centroids from which an initial partition is computed. There are also many methods to achieve this initial set [40], including random centroid selection [41]. The clustering performance may greatly depend on this [42], as *k*-means does not guarantee global optimality. For repeatability, we chose a max–min scheme to choose the two centroids [43]. The first record in the experimental database was taken as the centroid of one class, and the furthest to this one in terms of *d* was the initial centroid for the other class (max approach). The corresponding partition can then be obtained assigning each time series to its closest centroid (min approach). This way, the results will not alter across the experiments, at the expense of a possible suboptimal centroid selection [42].

At this point the *k*-Means iterations can take place. The centroids are replaced by an arithmetic partition average (stated another way, the histograms of all the time series assigned to a centroid are averaged), and the partition is built again using the new centroids. This process is repeated until a convergence criterion is met or a number of iterations is reached [44]. In this work, a fixed number of 10 iterations was used to keep the computational cost low and constant. This can also come at the expense of a loss of performance, but the goal of the present study was not to design a classifier, but to illustrate how the estimated motif probabilities as a feature vector can enhance the discriminating power of PE.

In general, the time complexity of the standard *k*-means clustering algorithm is O(lskm!), with *l* being the number of iterations, *s* being the number of objects (size of the dataset), *k* being the number of clusters and m! being the dimension of the input vector [45]. In each iteration, the distance to each centroid from each object has to be computed. That makes sk distances. As each distance entails an arithmetic operation with each vector component, then each time a partition is computed needs at least skm! operations. As this is repeated at each iteration, the global computational cost can be roughly estimated as O(lskm!), as stated. However, this is a very conservative upper bound for the algorithm complexity since this a worst case approach [46]. Moreover, further optimisations can be applied [47,48].

In practice, l<<s, k<<s and m<<s, being the number of time series *s* the main factor influencing the computational cost. However, m! grows very fast, and the clustering task may soon become a high-dimensional clustering problem [49]. Fortunately, as already demonstrated in [23], only a few features, namely, histogram bins in the present paper, are responsible for differences among classes (or many bins are just 0), and therefore, more efficient sparse clustering algorithm versions can be used instead [50]. Besides, as *m* grows, the compensation probability among histogram bins decreases, as occurs for the sum of several dice [51] when the number of dice increases.

The performance of the method was quantified in terms of classification accuracy in all cases, given by the percentage of correctly classified times series. Further details of the *k*-means algorithm can be found in many scientific papers, such as in [32,52]. Improvements to the standard *k*-means algorithm used in this paper are also extensively described elsewhere [53,54,55,56].

### 2.3. Hidden Markov Models

HMM are stochastic processes that can be used to generate symbol sequences [57]. They consist of a number of nodes representing *M* states, $Q=\left\{q_{0},q_{1},\cdots ,q_{M-1}\right\}$. These nodes are interconnected by links related to a transition probability between any two states, in other words, the probability of being at state $q_{j}$ at time t+1 if we were at state $q_{i}$ at previous time *t*. In principle, any HMM includes a bidimensional transition probability matrix $A=\left\{a_{ij}\right\}$ with *M* rows and *M* columns, in its simplest and more generic case. Thus, $a_{ij}=p\left[State_{t+1}=q_{j}|State_{t}=q_{i}\right]$. There also exists another set of probabilities of emitting a specific symbol at each state $q_{i}$; but, in this work, each state generates a single entire motif (ordinal pattern) with probability 1, and therefore these probabilities will not be considered.

To discover the values of all the parameters involved in HMMs, three problems have to be solved first [57]:

- To compute the probability of observing a certain input vector (Evaluation).
- To find a state transition sequence that maximizes the probability of a certain input vector (Generation).
- To induce a model that maximises the probability of a certain input vector (Learning).

However, this study uses HMM only for generation of synthetic time series based on constrains related to possible ordinal patterns. Moreover, the generation will be used in the opposite direction. That is, the input to the HMM will be the transition probabilities between states, and with a random excitation, a sequence of ordinal patterns will be obtained to generate a synthetic time series accordingly.

**Figure 2:**
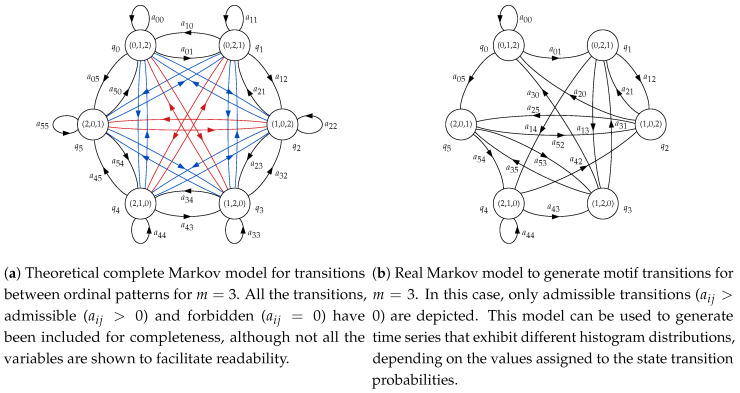
Basic Markov models for consecutive ordinal patterns in a time series for m=3.

For m=3, the HMM used will have six states, from $q_{0}$ to $q_{5}$. The correspondence between states and ordinal patterns will be $q_{0}\leftarrow \left(0,1,2\right)$, $q_{1}\leftarrow \left(0,2,1\right)$, $q_{2}\leftarrow \left(1,0,2\right)$, $q_{3}\leftarrow \left(1,2,0\right)$, $q_{4}\leftarrow \left(2,1,0\right)$ and $q_{5}\leftarrow \left(2,0,1\right)$. This means that if, at time *t*, for example, state $q_{3}$ is reached, the ordinal pattern to consider must be (1,2,0), and therefore a synthetic random sample subsequence $x_{i}^{3}$ following this order will have to be generated and appended to the complete time series. The topology will be ergodic, considering state transition probabilities $a_{ij}$ between any two states in general. The structure of this model is shown in Figure 2a. The initial state of the HMM, $State_{t=0}=q_{i}$, will be chosen randomly.

However, not all the transitions between ordinal patterns are possible, as there are two samples overlapping between consecutive subsequences. Therefore, the order and values of the last two samples of subsequence $x_{i-1}^{3}$ have to be the same for the two first samples of $x_{i}^{3}$. In practical terms, these forbidden transitions will be represented by a specific $a_{ij}=0$, in contrast to the possible or admissible transitions, where $a_{ij}>0$. This will be explained in more detail in next section. The resulting model applying these constraints is shown in Figure 2b, and this is the model that will be used to generate synthetic time series.

### 2.4. Experimental Dataset

The experimental dataset was composed of synthetic and real-life records. The synthetic time series were devised to fine tune the content in terms of ordinal patterns and enable the study of specific effects. This way, the study would be capable of unveiling the possible weaknesses of the methods assessed. The real-life dataset was chosen with the objective of translating into practical terms the lessons learnt with the synthetic data.

#### 2.4.1. Synthetic Dataset

The creation of a controlled set of ordinal patterns is not a straightforward task. Even for low *m* values, there are many constraints that have to be taken into account. For example, as described in [58], and also stated above, not all the transitions between consecutive motifs in PE are possible. These transitions are summarised for m=3 in Table 1. The rationale of this behaviour is that consecutive subsequences overlap. Namely, given an initial subsequence $x_{i}=\left\{x_{i},x_{i+1},x_{i+2},\ldots ,x_{i+m-1}\right\}$, the next consecutive subsequence $x_{i+1}$ shares all the values with $x_{i}$ except the first one, as $x_{i+1}=\left\{x_{i+1},x_{i+2},x_{i+3},\ldots ,x_{i+m}\right\}$. In other words, the last m-1 samples of $x_{i}$ are the first m-1 samples of $x_{i+1}$. Consequently, the order found for $\left\{x_{i+1},x_{i+2},x_{i+3},\ldots ,\right\}$ has to be the same both in $x_{i}$ and in $x_{i+1}$. For example, let $x_{i}^{3}=\left\{2.13,4.56,6.29\right\}$ for m=3, with an associated ordinal pattern $\pi_{i}^{3}=\left\{0,1,2\right\}$. The next subsequence will have to be of the form $x_{i+1}=\left\{4.56,6.29,\lambda \right\}$.

There are three possible regions where the value of λ can fall: λ<min(4.56,6.29), min(4.56,6.29)<λ<max(4.56,6.29) or λ>max(4.56,6.29) (equal values are not included to avoid ties [59,60]). In the first case, it would correspond to an ordinal pattern $\pi_{i}^{3}=\left\{2,0,1\right\}$; in the second case, to $\pi_{i}^{3}=\left\{0,2,1\right\}$ and; in the last case, to $\pi_{i}^{3}=\left\{0,1,2\right\}$, being any other ordinal pattern impossible to achieve. Analytically, this could have been anticipated from all the motifs that respect the relative order of (4.56,6.29)$(\left\{0,1\right\}$): $\left\{2,0,1\right\},\left\{0,2,1\right\}$ and $\left\{0,1,2\right\}$. Although more complex, this reasoning can be extrapolated for m>3.

This behaviour of the transitions between consecutive motifs fits very well into a HMM [57]. The synthetic time series to be created can be considered an overlapping sequence of random values whose order is given by a motif from a finite and known alphabet. This knowledge of the motifs, their sequence and their probabilities of occurrence, enables the definition of a discrete time invariant stochastic model able to generate synthetic sequences. These sequences can exhibit a desired statistical distribution of motifs given the appropriate input excitation and state transition probabilities.

Of the initially possible 36 state transition probabilities, those 18 corresponding to forbidden transitions were permanently fixed to 0 in the HMM. The other 18 non-null state transition probabilities were grouped according to the state of origin of the transition. Thus, $a_{00}$, $a_{01}$ and $a_{05}$ were considered a group of state transition probabilities from state $q_{0}$, $a_{12}$, $a_{13}$ and $a_{14}$ for $q_{1}$, $a_{20}$, $a_{21}$ and $a_{25}$ for $q_{2}$, $a_{30}$, $a_{31}$ and $a_{35}$ for $q_{3}$, $a_{42}$, $a_{43}$, and $a_{44}$ for $q_{4}$, and finally, $a_{52}$, $a_{53}$, $a_{54}$ for $q_{5}$. To avoid configuring 18 probabilities for each realisation of the experiments with synthetic records, the probabilities were set using these groups of three. For example, when stated that probabilities were set as $a_{ij}=\left\{\frac{1}{10},\frac{2}{10},\frac{7}{10}\right\}$, $a_{12}=\frac{1}{10}$, $a_{13}=\frac{2}{10}$, $a_{14}=\frac{7}{10}$, $a_{20}=\frac{1}{10}$, $a_{21}=\frac{2}{10}$, $a_{25}=\frac{7}{10}$ and so on.

**Figure 3:**
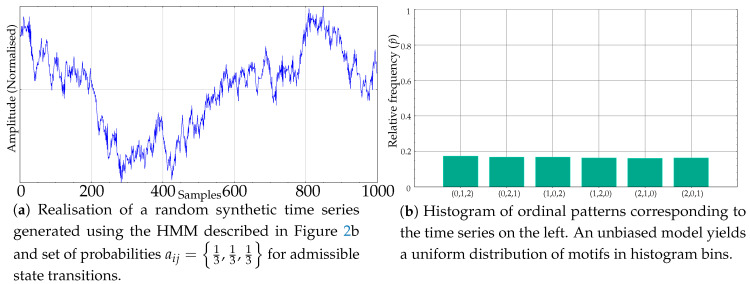
Example of a synthetic record generated using an unbiased HMM and its resulting uniform motif relative frequencies.

Using the model in Figure 2b, and a suitable random excitation, the time series of any length with subsequences whose ordinal patterns are equally or unequally probable can be generated easily. As an example, see Figure 3a, whose histogram in Figure 3b resembles that of a random time series, like the one shown in Figure 1d. The random excitation determines the final transitions that take place at each *t* from an initial random state. The path followed, in terms of $q_{i}$, chains the ordinal patterns emitted, and from these ordinal patterns, subsequences of time series values are also randomly generated, ensuring they overlap with the previous ones and abide by the order emitted. These subsequences are generated ensuring also that ties do not take place, and therefore a clear falling or rising trend may be exhibited, depending on the dominant ordinal pattern, if any.

**Figure 4:**
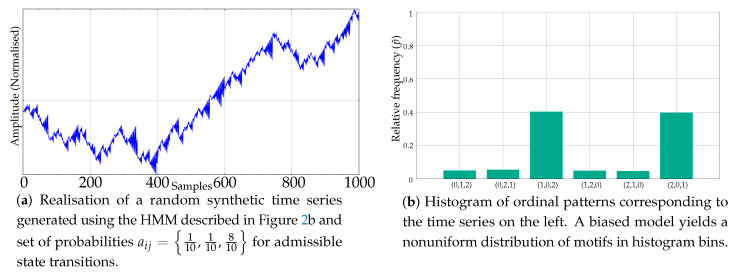
Example of a synthetic record generated using a biased version of the Markov model and its resulting nonuniform motif relative frequencies.

If the transition probabilities are not equal, what we obtain is a biased model instead that results in a nonuniform histogram of ordinal patterns. For example, Figure 4a shows a synthetic time series resulting from probabilities $a_{ij}=\left\{\frac{1}{10},\frac{1}{10},\frac{8}{10}\right\}$. Its histogram is depicted on its right, in Figure 4b.

In the experiments, two classes of synthetic records were generated using the approach described, termed Class A and Class B, with two sets of different probabilities. A total of 100 sequences of length 1000 samples were randomly created for each class.

#### 2.4.2. Real Dataset

**Figure 5:**
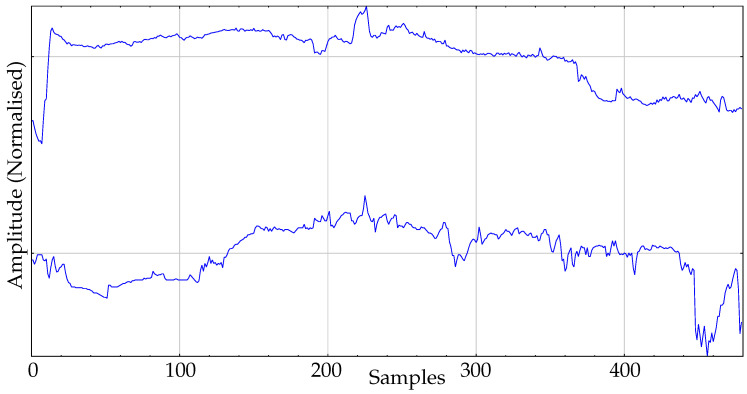
Example of records from the two classes of body temperature data. Top: Pathological. Bottom: Control.

The real dataset used in the experiments is composed of body temperature records. This dataset is the same previously used in other works [16], where additional details can be found. It contains 30 records and two classes. One class corresponds to a group of healthy individuals monitored during their daily activities: the control group. The size of this group is 16. The second group, the pathological group, contains 14 records of admitted patients at the Internal Medicine ward of the Teaching Hospital of Móstoles, Madrid. These patients developed a fever at least 24h before the temperature data acquisition started. An example of records of each class is shown in Figure 5.

## 3. Experiments and Results

The discriminating power of PE has been well demonstrated in a number of publications [16,24,60,61,62]. As a single feature, using a threshold, it has been possible to successfully classify a disparity of records of different types.

Initially, the experiments were devised to assess the sensitivity of PE in terms of histogram differences that led to PE differences as well. The two class synthetic records used in these experiments where generated using unbiased and biased versions of the HMM described above. The unbiased model using equal transition probabilities $a_{ij}=\left\{\frac{1}{3},\frac{1}{3},\frac{1}{3}\right\}$ (Class A) and biased versions, with unbalanced probabilities defined by the relationship $a_{ij}=\left\{\alpha ,\alpha ,1-2\alpha \right\}$, with 0≤α≤0.5 (Class B). In this case, PE was expected to achieve a classification accuracy close to 100% using records from the synthetic dataset, given that Class A and B are very different.

The results of this test are shown in Table 2. Class A was always fixed to an unbiased version (only the initial state was chosen at random), compared against different probability combinations featuring a biased histogram for Class B. The results confirmed that these two classes were easily separable, with poor classification performance only for values of α really close to the unbiased version of $\frac{1}{3}$. The classification accuracy is given as the average value and standard deviation of the 10 realisations of each experiment, where at least the initial state could vary.

In the next set of experiments, transition probabilities were more varied for both synthetic classes. The main objective in this case was to find histograms with similar amplitude bins but at different locations (motifs) in order to assess the possible PE loss of discriminating power. Note that the relationship between probabilities and motifs is given by the graphical structure of the model shown in Figure 2b. In other words, an asymmetry in the transition probabilities does not necessarily entail the same asymmetry in the histogram bins, since the state (motif emitter) can be reached from multiple paths. These results are shown in Table 3.

**Figure 6:**
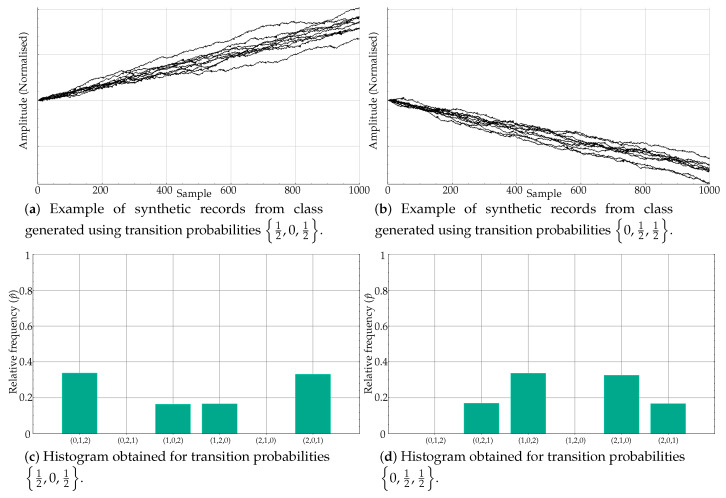
Equivalent histograms from the PE point of view despite clear differences in ordinal pattern distribution. When the estimated probability is computed, the PE measure is more or less the same since the differences in bin locations are lost, only amplitudes matter.

Some of the experiments in Table 3 are representative of the PE weakness addressed in the present paper: very similar relative frequencies in different motifs lead to the same PE value, and therefore classes become indistinguishable from a classification perspective. Specifically, this is the case generated by means of transition probabilities $\left\{\frac{1}{2},0,\frac{1}{2}\right\}$ and $\left\{0,\frac{1}{2},\frac{1}{2}\right\}$. The time series generated using these probabilities are shown in Figure 6a,b, respectively. Despite having a very different motif distribution, the $\hat{p}$ values are almost identical, at different positions, yielding similar PE values.

For example, for $\left\{\frac{1}{2},0,\frac{1}{2}\right\}$, the main motifs were (0,1,2) and (2,0,1), followed by (1,0,2) and (1,2,0). Motifs (0,2,1) and (2,1,0) were negligible, as depicted in Figure 6c. On the other hand, for $\left\{0,\frac{1}{2},\frac{1}{2}\right\}$, the main motifs were (1,0,2) and (2,1,0), then (0,2,1) and (2,1,0), with no ordinal patterns (0,1,2) and (1,2,0) (see Figure 6d). However, bin amplitudes were very similar, with an equal mapping on a single PE value.

**Figure 7:**
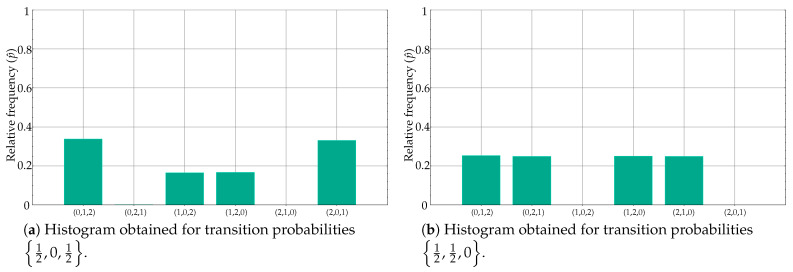
Contrary to case depicted in Figure 6, differences in ordinal pattern distribution are supported by differences in histogram amplitudes, which results in significant PE differences as well.

However, the results in Table 3 for $\left\{\frac{1}{2},0,\frac{1}{2}\right\}$ and $\left\{\frac{1}{2},\frac{1}{2},0\right\}$, despite having a similar transition probability set of values, are completely different from an histogram perspective, and that is why the classification accuracy was 100%. This is very well illustrated in Figure 7a,b.

Figure 6 and Figure 7 very well summarise what can occur in a real case using PE as the distinguishing feature of a classification procedure (or any other similar mapping approach). Although the case in Figure 7 is the most frequent case, it is worth exploring alternatives to deal with poor time series classification performances based on PE, as this study proposes. Along this line, the results shown in Table 4 correspond to the same experiments as in Table 2 and Table 3, but using the relative frequencies instead and the clustering algorithm as described in Section 2.2.

The classification performance achieved in the experiments in Table 4 were clearly superior to those achieved using only PE, as hypothesised. For those cases where PE had a high discriminating power already, using the raw estimated probabilities vector and the *k*-Means clustering algorithm, such power was maintained or even slightly increased. For the specific case described in Figure 6, where PE failed as commented above, this time the performance achieved an expected 100%, given the differences in the motif distribution. Only when there were no significant differences, with equal transition probabilities, both methods obviously failed.

The experiments were repeated using real body temperature time series from the Control and Pathological datasets described in Section 2.4.2. The relative frequencies of each motif for the first subset are numerically shown in Table 5 for each record. The same for pathological records, in Table 6.

Temperature records of the experimental dataset exhibit the same behaviour than synthetic records with transition probabilities $\left\{\frac{1}{2},0,\frac{1}{2}\right\}$ and $\left\{0,\frac{1}{2},\frac{1}{2}\right\}$. Using PE as the single classification feature, it was not possible to find differences between classes, with a nonsignificant accuracy of 60%. With the clustering and probability vector approach, this accuracy raised up to the 90%, with only three misplaced time series (19 objects assigned to the Control class, 16 really Control ones and the three errors, and 11 to the pathological one). The final centroids for each class were $\left\{0.3812,0.0928,0.0992,0.1166,0.1885,0.1229\right\}$ and $\left\{0.3667,0.0931,0.0976,0.1177,0.2035,0.1218\right\}$.

Although it was not the objective of the present study to design a classifier, to better support the results using the estimated probabilities instead of PE, a leave-one-out (LOO) [63] classification analysis was conducted on the temperature data. A total of 100 realisations are used. In each realisation, a record from each class (with replacement) was randomly omitted in the *k*-means analysis. Then, the resulting centroids were used for classification of the omitted records using a nearest neighbour approach. The number of errors in this case were 18, that is, 18 records of the Pathological group were incorrectly classified as Control records, whereas no Control record was misclassified as Pathological. Therefore, the global accuracy using LOO and the probability vectors of the temperature records was 82%.

## 4. Discussion

The initial results in Table 2 give a sense of the PE general behaviour, in terms of discriminating power. For very significantly different transition probabilities, the classification performance easily reaches 100%. As these probabilities become closer, the error increases, and obviously, for the same models, it is impossible to distinguish between the two classes (54.8%). It is important to note that the probability window for which performance is not 100% is very narrow, from 0.33 to 0.3 accuracy goes from 54.8% up to 96.1%, which, in principle, confirms the high discriminating power of PE.

When both state transition probabilities are biased, the classification results are not 100% so often, as Table 3 illustrates. Moreover, although the probabilities are the same but in a different order, they result in significant differences in the histograms translated into a still high classification accuracy due to the asymmetry of the model (Figure 2b). In a few transition probabilities cases, as for $\left\{\frac{1}{2},0,\frac{1}{2}\right\}$ and $\left\{0,\frac{1}{2},\frac{1}{2}\right\}$, these histogram differences can be numerically compensated and lose the discriminating capability, as hypothesised in this study.

Table 4 demonstrates this point by applying a clustering algorithm to the set of relative frequency values instead of the resulting PE value. For all the theoretically separable cases, this scheme yields a performance of at least 96%, and, in all cases, the performance is higher than that achieved using only PE. It is especially significant the $\left\{\frac{1}{2},0,\frac{1}{2}\right\}$ and $\left\{0,\frac{1}{2},\frac{1}{2}\right\}$ case, a clear representative of the possible detrimental effects of mapping to a single feature. From a nonsignificant classification accuracy of 55%, the clustering approach achieves a 100% accuracy, since the class differences are very apparent in the set of relative frequencies (Figure 6c,d). Obviously, all methods fail when the state transition probabilities are the same (51.1% accuracy in this case for $\frac{1}{3}$ probabilities). Although with PE the result was 54.8% accuracy, it should not be interpreted as an improvement over the 51.1%, since both results were not significant, and correspond to a plain random guess that can exhibit a minor bias due to the limited number of realisations, 10 in this case.

Table 5 and Table 6 demonstrate, with real data, the possible detrimental impact on PE discriminating power mapping all the relative frequencies on a single scalar may exert, confirming what synthetic records already showed. The numerical values listed enable a detailed comparison of each motif contribution to the possible differences between classes. The main differences take place for patterns (0,1,2) and (2,1,0), at the first and fifth data columns, respectively. When using PE values, these differences become less noticeable, and that is why it is not possible to separate significantly the Control and Pathological groups of these records. Moreover, these relative frequencies go in opposite directions, that is, they are greater for the Control group in the first case (0.3905>0.3584) and smaller in the second case (0.1813<0.2087), with relatively small standard deviations, and therefore compensation is more likely. It is also important to note that the final clustering centroids very well captured the histogram structure of both classes.

The LOO analysis provided a more realistic picture of the possible performance if a classifier had to be designed for the experimental dataset of temperature records. The accuracy went down slightly, from 90% using the entire dataset, down to 82% when a record of each class was omitted in the centroid computation. Anyway, this is still a very high classification accuracy, and it is important to note that PE alone was unable to find significant differences in this case, even when using all the records.

The computational cost using m! features instead of a single PE value was obviously expected to be higher. Therefore, a trade–off between accuracy and execution time should be considered. Using a Windows© 8 computer with an Intel Core i7-4720HQ© 2.60GHz processor, with 16GB of RAM and C++ programming language, the execution time for the 100 LOO iterations using temperature records was 5.92s for m=3, 21.1s for m=4 and 104.1s for m=5, without any algorithm optimisation. With a single feature, PE, the execution time was 2.48s.

The embedded dimension *m* is also related to the amount of information captured by a subsequence. A multiscale approach, with different time scales for PE [64,65], could also contribute to gain more insights into the dynamics of the time series. In this regard, it has been demonstrated that higher values of *m* frequently provide more discriminating power [24,60], as well as an optimal set of time delays [66]. However, using the approach proposed, it would be first necessary to assess the significance of the m! features, even for each time delay, to keep the computational cost reasonably low.

## 5. Conclusions

The distinguishing power of PE is very high and is sufficient for many time series classification applications. PE has been successfully employed as a single feature in a myriad of technological and scientific fields.

However, sometimes the distinguishing features embedded in the histogram bins can become blurred when Equation 3 is applied. In order to avoid this problem, before discarding PE as the classification feature, or any other measure based on this kind of many-to-one mapping approach, we propose to look at the previous step of the PE algorithm and analyse the discriminating power of the relative frequencies instead.

The experimental set had to be chosen with this problem in mind. To control, up to a certain extent, the distribution of ordinal patterns in the time series used, we first developed a method based on HMM to create synthetic time series that satisfied certain motif emission constraints, and caused PE to fail at least in some cases. In addition, real-time series and body temperature records were also included, as they also exhibited this same problem under study.

The use of a vector of features instead of a single value can be seen as a typical multifeature extraction stage of a pattern recognition task. Almost any clustering algorithm is perfectly suited to deal with this task. For illustrative purposes only—not for optimisation—we applied a classical k-Means algorithm and a standard configuration.

The results using this approach confirmed, for both datasets, that using the original histogram values, the discriminating power of PE can be enhanced. As a consequence, we propose, when possible, to analyse the information provided by each histogram bin jointly, as is the case in the present study, or separately, in a kind of feature selection analysis, as in [23], to maximise the information provided by ordinal pattern distribution beyond the scalar PE value. This approach can be arguably be exported to many other methods, and open a new line of research combining event-counting metrics with pattern recognition or machine learning algorithms.

Obviously, if there are no differences at the histogram level; for example, when temporal correlations are the same, any method based on such information will fail, that is, both PE and the method proposed. This scenario can be illustrated trying to distinguish time series with random Gaussian or uniform amplitude distributions. Despite the amplitude differences, it was not possible to distinguish between both classes using PE or the method proposed. For such cases, we conducted a few additional tests using measures better suited to amplitude differences in order to propose alternatives to overcome this drawback. The most straightforward approach is to use methods such as ApEn or SampEn, both amplitude-based, which achieved a classification accuracy close to 100% using Gaussian and uniform amplitude distributions for classes. A solution more related to the present paper was to use a PE derivative that included amplitude information [24]. In this regard, Weighted Permutation Entropy [67] also achieved a very high classification accuracy, well above 80%.

## Figures and Tables

**Table 1:** Admissible and forbidden transitions between consecutive motifs for m=3.

Initial Motif	Admissible Next Motifs	Forbidden Next Motifs
$\left\{0,1,2\right\}$	$\left\{0,1,2\right\}$	$\left\{1,2,0\right\}$
	$\left\{0,2,1\right\}$	$\left\{2,1,0\right\}$
	$\left\{2,0,1\right\}$	$\left\{1,0,2\right\}$
$\left\{0,2,1\right\}$	$\left\{1,2,0\right\}$	$\left\{0,1,2\right\}$
	$\left\{2,1,0\right\}$	$\left\{0,2,1\right\}$
	$\left\{1,0,2\right\}$	$\left\{2,0,1\right\}$
$\left\{1,2,0\right\}$	$\left\{0,1,2\right\}$	$\left\{1,2,0\right\}$
	$\left\{0,2,1\right\}$	$\left\{2,1,0\right\}$
	$\left\{2,0,1\right\}$	$\left\{1,0,2\right\}$
$\left\{2,0,1\right\}$	$\left\{1,2,0\right\}$	$\left\{0,1,2\right\}$
	$\left\{2,1,0\right\}$	$\left\{0,2,1\right\}$
	$\left\{1,0,2\right\}$	$\left\{2,0,1\right\}$
$\left\{2,1,0\right\}$	$\left\{1,2,0\right\}$	$\left\{0,1,2\right\}$
	$\left\{2,1,0\right\}$	$\left\{0,2,1\right\}$
	$\left\{1,0,2\right\}$	$\left\{2,0,1\right\}$
$\left\{1,0,2\right\}$	$\left\{0,1,2\right\}$	$\left\{1,2,0\right\}$
	$\left\{0,2,1\right\}$	$\left\{2,1,0\right\}$
	$\left\{2,0,1\right\}$	$\left\{1,0,2\right\}$

**Table 2:** Classification performance of permutation entropy (PE) as a single feature when one class of synthetic records was generated using a balanced set of probabilities, and the other class using a varied set of probabilities.

Class A	Class B	Classification
Transition Probabilities	Transition Probabilities	Accuracy (%)
$\left\{\frac{1}{3},\frac{1}{3},\frac{1}{3}\right\}$	$\left\{\frac{1}{10},\frac{1}{10},\frac{8}{10}\right\}$	100±0
$\left\{\frac{1}{3},\frac{1}{3},\frac{1}{3}\right\}$	$\left\{\frac{1}{9},\frac{1}{9},\frac{7}{9}\right\}$	100±0
$\left\{\frac{1}{3},\frac{1}{3},\frac{1}{3}\right\}$	$\left\{\frac{1}{8},\frac{1}{8},\frac{6}{8}\right\}$	100±0
$\left\{\frac{1}{3},\frac{1}{3},\frac{1}{3}\right\}$	$\left\{\frac{1}{7},\frac{1}{7},\frac{5}{7}\right\}$	100±0
$\left\{\frac{1}{3},\frac{1}{3},\frac{1}{3}\right\}$	$\left\{\frac{1}{6},\frac{1}{6},\frac{4}{6}\right\}$	100±0
$\left\{\frac{1}{3},\frac{1}{3},\frac{1}{3}\right\}$	$\left\{\frac{1}{5},\frac{1}{5},\frac{3}{5}\right\}$	100±0
$\left\{\frac{1}{3},\frac{1}{3},\frac{1}{3}\right\}$	$\left\{\frac{2}{7},\frac{2}{7},\frac{3}{7}\right\}$	99.5±0.57
$\left\{\frac{1}{3},\frac{1}{3},\frac{1}{3}\right\}$	$\left\{\frac{4}{13},\frac{4}{13},\frac{5}{13}\right\}$	87.4±1.88
$\left\{\frac{1}{3},\frac{1}{3},\frac{1}{3}\right\}$	$\left\{\frac{1}{3},\frac{1}{3},\frac{1}{3}\right\}$	54.8±1.20
$\left\{\frac{1}{3},\frac{1}{3},\frac{1}{3}\right\}$	$\left\{\frac{3}{10},\frac{3}{10},\frac{4}{10}\right\}$	96.1±1.65
$\left\{\frac{1}{3},\frac{1}{3},\frac{1}{3}\right\}$	$\left\{\frac{1}{4},\frac{1}{4},\frac{2}{4}\right\}$	100±0

**Table 3:** Classification performance of PE as a single feature when both synthetic classes are generated using a biased set of probabilities.

Class A	Class B	Classification
Transition Probabilities	Transition Probabilities	Accuracy (%)
$\left\{\frac{12}{20},\frac{5}{20},\frac{3}{20}\right\}$	$\left\{\frac{3}{20},\frac{5}{20},\frac{12}{20}\right\}$	87.5±2.87
$\left\{\frac{13}{20},\frac{4}{20},\frac{3}{20}\right\}$	$\left\{\frac{3}{20},\frac{4}{20},\frac{13}{20}\right\}$	95.3±0.83
$\left\{\frac{10}{20},\frac{6}{20},\frac{4}{20}\right\}$	$\left\{\frac{9}{20},\frac{3}{20},\frac{8}{20}\right\}$	85.4±1.43
$\left\{\frac{1}{2},0,\frac{1}{2}\right\}$	$\left\{0,\frac{1}{2},\frac{1}{2}\right\}$	55.0±2.89 (Figure 6)
$\left\{\frac{1}{2},0,\frac{1}{2}\right\}$	$\left\{\frac{1}{2},\frac{1}{2},0\right\}$	100±0 (Figure 7)

**Table 4:** Classification performance achieved using the 6 estimated probability values as a feature vector instead of the single PE value, for the previous experiments.

Class A	Class B	Classification
Transition Probabilities	Transition Probabilities	Accuracy (%)
$\left\{\frac{1}{3},\frac{1}{3},\frac{1}{3}\right\}$	$\left\{\frac{1}{10},\frac{1}{10},\frac{8}{10}\right\}$	100±0
$\left\{\frac{1}{3},\frac{1}{3},\frac{1}{3}\right\}$	$\left\{\frac{1}{5},\frac{1}{5},\frac{3}{5}\right\}$	100±0
$\left\{\frac{1}{3},\frac{1}{3},\frac{1}{3}\right\}$	$\left\{\frac{2}{7},\frac{2}{7},\frac{3}{7}\right\}$	99.7±0.51
$\left\{\frac{1}{3},\frac{1}{3},\frac{1}{3}\right\}$	$\left\{\frac{4}{13},\frac{4}{13},\frac{5}{13}\right\}$	96.0±2.23
$\left\{\frac{1}{3},\frac{1}{3},\frac{1}{3}\right\}$	$\left\{\frac{1}{3},\frac{1}{3},\frac{1}{3}\right\}$	51.1±1.74
$\left\{\frac{1}{3},\frac{1}{3},\frac{1}{3}\right\}$	$\left\{\frac{3}{10},\frac{3}{10},\frac{4}{10}\right\}$	99.0±0.57
$\left\{\frac{12}{20},\frac{5}{20},\frac{3}{20}\right\}$	$\left\{\frac{3}{20},\frac{5}{20},\frac{12}{20}\right\}$	100±0
$\left\{\frac{13}{20},\frac{4}{20},\frac{3}{20}\right\}$	$\left\{\frac{3}{20},\frac{4}{20},\frac{13}{20}\right\}$	100±0
$\left\{\frac{10}{20},\frac{6}{20},\frac{4}{20}\right\}$	$\left\{\frac{9}{20},\frac{3}{20},\frac{8}{20}\right\}$	100±0
$\left\{\frac{1}{2},0,\frac{1}{2}\right\}$	$\left\{0,\frac{1}{2},\frac{1}{2}\right\}$	100±0
$\left\{\frac{1}{2},0,\frac{1}{2}\right\}$	$\left\{\frac{1}{2},\frac{1}{2},0\right\}$	100±0

**Table 5:** Individual results for the estimated frequencies for each motif using the control records.

Record	(0,1,2)	(0,2,1)	(1,0,2)	(1,2,0)	(2,1,0)	(2,0,1)
Control00	0.4058	0.0836	0.0941	0.1255	0.1548	0.1359
Control01	0.5376	0.0899	0.0836	0.0878	0.1192	0.0815
Control02	0.3849	0.0753	0.0836	0.1025	0.2426	0.1108
Control03	0.3870	0.0732	0.0878	0.1129	0.2092	0.1297
Control04	0.3242	0.1276	0.1255	0.1171	0.1903	0.1150
Control05	0.3598	0.0962	0.0983	0.1213	0.2008	0.1234
Control06	0.3912	0.0815	0.0648	0.1401	0.1987	0.1234
Control07	0.3410	0.1004	0.1317	0.0815	0.2322	0.1129
Control08	0.3368	0.0941	0.0983	0.1171	0.2301	0.1234
Control09	0.3159	0.1171	0.1255	0.1234	0.1841	0.1338
Control10	0.4560	0.0920	0.0962	0.1129	0.1276	0.1150
Control11	0.3556	0.1004	0.1171	0.1213	0.1694	0.1359
Control12	0.5334	0.0732	0.0753	0.0899	0.1380	0.0899
Control13	0.2615	0.1066	0.1234	0.1317	0.2301	0.1464
Control14	0.4058	0.0836	0.0941	0.1255	0.1548	0.1359
Control15	0.4518	0.0774	0.0878	0.1276	0.1192	0.1359
Mean	0.3905	0.0920	0.0992	0.1149	0.1813	0.1218
StdDev	0.0751	0.0157	0.0198	0.0165	0.0421	0.0174

**Table 6:** Individual results for the estimated frequencies for each motif using the pathological records.

Record	(0,1,2)	(0,2,1)	(1,0,2)	(1,2,0)	(2,1,0)	(2,0,1)
Pathological00	0.4054	0.0838	0.0920	0.1085	0.1938	0.1161
Pathological01	0.3260	0.0960	0.1036	0.1207	0.2244	0.1290
Pathological02	0.4496	0.0659	0.0683	0.1163	0.1822	0.1175
Pathological03	0.3405	0.0980	0.0926	0.1089	0.2534	0.1062
Pathological04	0.2191	0.1271	0.1348	0.1374	0.2347	0.1465
Pathological05	0.2736	0.0979	0.1083	0.1348	0.2407	0.1444
Pathological06	0.3805	0.0979	0.0992	0.1204	0.1793	0.1224
Pathological07	0.3331	0.0950	0.0950	0.1347	0.2072	0.1347
Pathological08	0.4361	0.0711	0.0660	0.1219	0.1873	0.1174
Pathological09	0.3103	0.1065	0.1019	0.1287	0.2283	0.1241
Pathological10	0.4342	0.0756	0.0919	0.0968	0.1881	0.1131
Pathological11	0.3447	0.1224	0.1155	0.1265	0.1716	0.1190
Pathological12	0.3590	0.0972	0.1072	0.1054	0.2154	0.1154
Pathological13	0.4056	0.0838	0.0943	0.0950	0.2154	0.1056
Mean	0.3584	0.0942	0.0979	0.1183	0.2087	0.1222
StdDev	0.0657	0.0174	0.0173	0.0137	0.0255	0.0125
